# AnaLysis of Expression on human chromosome 21, ALE-HSA21: a pilot integrated web resource

**DOI:** 10.1093/database/bau009

**Published:** 2014-02-25

**Authors:** Margherita Scarpato, Roberta Esposito, Daniela Evangelista, Marianna Aprile, Maria Rosaria Ambrosio, Claudia Angelini, Alfredo Ciccodicola, Valerio Costa

**Affiliations:** ^1^Institute of Genetics and Biophysics ‘Adriano Buzzati-Traverso’, National Research Council, Naples, Italy, ^2^Department of Pharmaceutical Sciences, University of Salerno, National Research Council, Fisciano, Salerno, Italy, ^3^Istituto per le Applicazioni del Calcolo ‘Mauro Picone’, National Research Council, Naples, Italy and ^4^Department of Biochemistry and Biophysics, Second University of Naples (SUN), Naples, Italy

## Abstract

Transcriptome studies have shown the pervasive nature of transcription, demonstrating almost all the genes undergo alternative splicing. Accurately annotating all transcripts of a gene is crucial. It is needed to understand the impact of mutations on phenotypes, to shed light on genetic and epigenetic regulation of mRNAs and more generally to widen our knowledge about cell functionality and tissue diversity. RNA-sequencing (RNA-Seq), and the other applications of the next-generation sequencing, provides precious data to improve annotations' accuracy, simultaneously creating issues related to the variety, complexity and the size of produced data. In this ‘scenario’, the lack of user-friendly resources, easily accessible to researchers with low skills in bioinformatics, makes difficult to retrieve complete information about one or few genes without browsing a jungle of databases. Concordantly, the increasing amount of data from ‘omics’ technologies imposes to develop integrated databases merging different data formats coming from distinct but complementary sources. In light of these considerations, and given the wide interest in studying Down syndrome—a genetic condition due to the trisomy of human chromosome 21 (HSA21)—we developed an integrated relational database and a web interface, named ALE-HSA21 (AnaLysis of Expression on HSA21), accessible at http://bioinfo.na.iac.cnr.it/ALE-HSA21. This comprehensive and user-friendly web resource integrates—for all coding and noncoding transcripts of chromosome 21—existing gene annotations and transcripts identified *de novo* through RNA-Seq analysis with predictive computational analysis of regulatory sequences. Given the role of noncoding RNAs and untranslated regions of coding genes in key regulatory mechanisms, ALE-HSA21 is also an interesting web-based platform to investigate such processes. The ‘transcript-centric’ and easily-accessible nature of ALE-HSA21 makes this resource a valuable tool to rapidly retrieve data at the isoform level, rather than at gene level, useful to investigate any disease, molecular pathway or cell process involving chromosome 21 genes.

**Database URL**: http://bioinfo.na.iac.cnr.it/ALE-HSA21/

## Introduction

In the past years, transcriptome studies have largely shown the pervasive nature of transcription in living organisms (1–4). In particular, the GENCODE Consortium, by combining computational analysis, manual curation and experimental validations, has identified a plethora of new coding and noncoding RNA transcripts expressed in human cells (5). Altogether these studies demonstrate that a significant fraction of transcribed regions stands outside known genes and most of them undergo alternative splicing (AS). Therefore, defining a curated and validated gene annotation is difficult, even though crucial for several reasons. For instance, annotating all the transcripts of a given gene is strictly necessary to postulate the functional impact of nucleotide variations, particularly those located in genomic regions till now recognized as ‘nongenic’. The most striking evidence is mutations accounting for Mendelian disorders (6) or common variants associated to complex traits and diseases (7). Moreover, a correct annotation of mRNAs' untranslated regions (UTRs) would significantly help to investigate their role in the regulation of transcription and translation (8). Finally, identifying novel tissue or cell-specific coding and noncoding transcripts would significantly improve the knowledge of cell functionality.

Large-scale data sets produced by RNA-sequencing (RNA-Seq), an application of the next-generation sequencing, are revealing an optimal source to improve the accuracy of gene annotations (9). However, the variety, the complexity and the size of available data have exponentially increased, making them difficult to handle, analyze, store, share and integrate with those stored in existing databases (10). It is common for research groups—and/or large consortia—to annotate the same transcript, gene or protein isoform using different identifiers (id), often confounding unskilled users. For this reason, comparing and combining these data from different resources still represent a difficult task.

Another hurdle to overcome is data dispersion. For instance, having a complete landscape of regulatory molecules for a specific transcript of interest is challenging, as most of the available resources are difficult to browse without any expertise in bioinformatics, and often they appear too specialized. Several Web sites and tools have been specifically developed for the ‘sequence-based’ predictions of transcription factors' or microRNAs' binding sites but, to the best of our knowledge, such information has not been systematically integrated into already existing genomic resources. Such issues often limit the access to complete information for a transcript, gene or protein of interest without browsing different databases. Therefore, given the substantial lack of comprehensive and user-friendly Web sites for researchers or medical geneticists with low experience in bioinformatics, it is crucial to implement easy-to-use web resources. Concordantly, the massive production of ‘omics’ data makes urgent the need to develop integrated databases.

In light of these considerations, and given our interest in studying the expression of genes mapping on human chromosome 21 (HSA21)—whose triplication causes Down Syndrome (DS)—we developed an open-access integrated relational database and a web interface, ALE-HSA21 (AnaLysis of Expression on HSA21; http://bioinfo.na.iac.cnr.it/ALE-HSA21). Because RNA-Seq studies have indicated the need to investigate biological processes and disease mechanisms at isoform level, rather than at gene level, we designed our database as a ‘transcript-centric’ web portal.

It integrates—for all transcripts generated by AS from HSA21 genes—nucleotide sequences and exon/intron structures with data about regulatory sequences. The latter consist of computational predictions for canonical and noncanonical regulatory motifs within promoters, exons, introns and 3′ UTRs. Links to widely used genotype and phenotype databases are also integrated in ALE-HSA21, avoiding the user to browse different resources over the web. Finally, it contains a set of *de novo* discovered transcripts, identified through a robust computational analysis of our recently published RNA-Seq data sets (11).

This web portal clearly represents an estimable source of information for researchers and clinicians interested in studying DS. Given the proven role of HSA21 genes' dosage imbalance in DS clinical outcomes (11), having a comprehensive set of information about all HSA21 genes, and their alternative transcripts, is of major interest for researchers studying DS. The database is also a valuable resource for medical geneticists interested in other HSA21-related diseases. Disruption or alteration of binding sites for auxiliary splicing factors as well as of other gene regulatory regions—promoter and 3′ UTR—may affect mRNA transcription, processing and translation, in turn playing a pathological role.

This kind of information is available in ALE-HSA21 through an easily accessible web interface, designed to rapidly provide heterogeneous data in a scientifically rigorous—even though user-friendly—way.

## Materials and Methods

Links to the external databases, files and software (with version and parameters) are shown in Supplementary File S1.

### RNA-Seq data processing and gene annotations

Raw files containing short reads of 50 bp (.csfasta and .ual formats from SOLiD v3) originated by the massive-scale sequencing of endothelial progenitor cells (11), were filtered out for quality values, homology to adapters and rRNA sequences. Filtered reads were aligned with TopHat software version 1.1.4 (12) against the reference human genome (release hg19). Without providing annotated gene models, splice junctions were *de novo* determined by the software, and reported as a list in a standard tabular format (BED). Reads uniquely mapped on the reference genome—extracted from the output alignment file (BAM)—and junctions supported by at least three high-quality reads were used. For further analyses we extracted only HSA21 mapping reads. A coverage file (BEDGRAPH format) for HSA21 was created and loaded into an open-access session of University of California Santa Cruz (UCSC) Genome Browser, named ALE-HSA21. The track ‘Comprehensive Gene Annotation Set GENCODE Version 12’ was downloaded from Table Browser of UCSC in GTF format. The exact number and the category of HSA21 transcripts considered in our further analyses, and reported in ALE-HSA21, are listed in Supplementary Table S1. Manual curation was used—in some cases—to correctly assign, if any, the corresponding RefSeq ID to annotated GENCODE transcripts.

### Discovery of intronic and intergenic transcription

To identify potentially new exons and/or transcripts in introns and intergenic intervals, uniquely mapped reads were converted into sorted files in BED format using SAMtools and BEDtools (13, 14). Results were visualized in UCSC Genome Browser to assess the quality and the consistency of the mapping analysis. A customized workflow was built to extract the genomic coordinates of intronic and intergenic HSA21 transcripts from GENCODE annotation, as well as reads mapping within these regions. In detail, we divided intronic and intergenic intervals in windows of 200 and 500 bp, respectively, and we counted the number of reads falling within such windows. Putative windows were reported as potential new transcribed regions if the number of mapped reads was sufficiently large. Signal enrichment was evaluated by using a Poisson test similar in the spirit to MACS (15). The background (no-signal) intensity, λ, was independently estimated for intronic and intergenic regions by maximum likelihood approach. In particular, a subset of intergenic regions—>1 Mb and 10 kb distant from gene boundaries—was used to estimate λ_intergenic_. For the estimation of λ_intronic_ we used intronic intervals >300 kb and 1 kb away from exon boundaries. Poisson *P*-values computed in each window underwent Benjamini and Hochberg False Discovery Rate correction (16). Significant windows were merged in larger genomic regions when the distance among them was smaller or equal to the window size. Transcriptionally active regions were defined ‘high coverage peaks’ (HCPs). In addition, to assess the transcription upstream transcription start sites, and downstream the last nucleotide of annotated transcripts, we used windows of variable size (50–1000 bp). Coverage was evaluated by counting the number of reads mapping within these windows. We selected the transcripts to experimentally validate after the intersection with genomic coordinates of expressed sequence tags and gene predictions (AceView database), as well as by visual inspection in UCSC and Integrative Genomic Viewer.

### Computational analysis of splicing isoforms

The output of TopHat, i.e. the list of *de novo* determined splice junctions, was used to infer the evidence of unannotated isoforms generated by AS for each HSA21 gene. Each junction (j) consists of two connected blocks, left (L_j_) and right (R_j_). The length of each block, here defined as MOL_j_ and MOR_j_, is given by the Maximal Overhang (MO) over all reads mapping within L_j_ and R_j_, respectively (12).

The genomic coordinates (i.e. the starting and ending sites, S and E, respectively) of L_j_ and R_j_ were defined as follows:

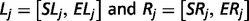

where

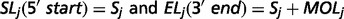

and

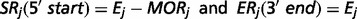

For the ‘plus’ strand, *S_j_* is the 5′ start genomic and *E_j_* is the 3′ end coordinate of *j*. *Vice versa*, for the ‘minus’ strand, *S_j_* is the 3′ end genomic coordinate of junction *j* and *E_j_* is the 5′ start coordinate of *j.* Thus, we independently intersected intervals L*_j_* and R*_j_* with the genomic coordinates of all exons of the HSA21 GENCODE v12 annotation by using the *intersectBed* function of BEDtools (13). Seven different categories of junctions were defined through this analysis (schematized in Supplementary Figure S1): (i) ‘annotated’ and (ii) ‘exon skipping’ if L*_j_* and R*_j_* map to consecutive or nonconsecutive exons on the same transcript, respectively; (iii) ‘only one side’, if only L*_j_* or R*_j_* map to one exon of a transcript and the other does not; (iv) ‘intra-exonic’ if both L*_j_* and R*_j_* map within the same exon; (v) ‘different transcripts’ if L*_j_* maps to an exon of a transcript and R*_j_* maps to an exon of another transcript belonging to the same gene; (vi) ‘trans-splicing’ if L*_j_* and R*_j_* map to exons of two different (adjacent) genes and (vii) ‘intronic/intergenic’ if both L*_j_* and R*_j_* do not map to any exon of an annotated transcript. Because a splice junction may belong to multiple categories (i.e. it may be ‘annotated’ for a given transcript but ‘exon skipping’ for another), we established a junction hierarchy (from category 1 to 7) in which junctions assigned to a category cannot be assigned to the next one.

Finally, each HSA21 GENCODE transcript was associated to a known official gene symbol to identify potentially new splice isoforms.

### Computational prediction of regulatory sequences

**Figure 1.**
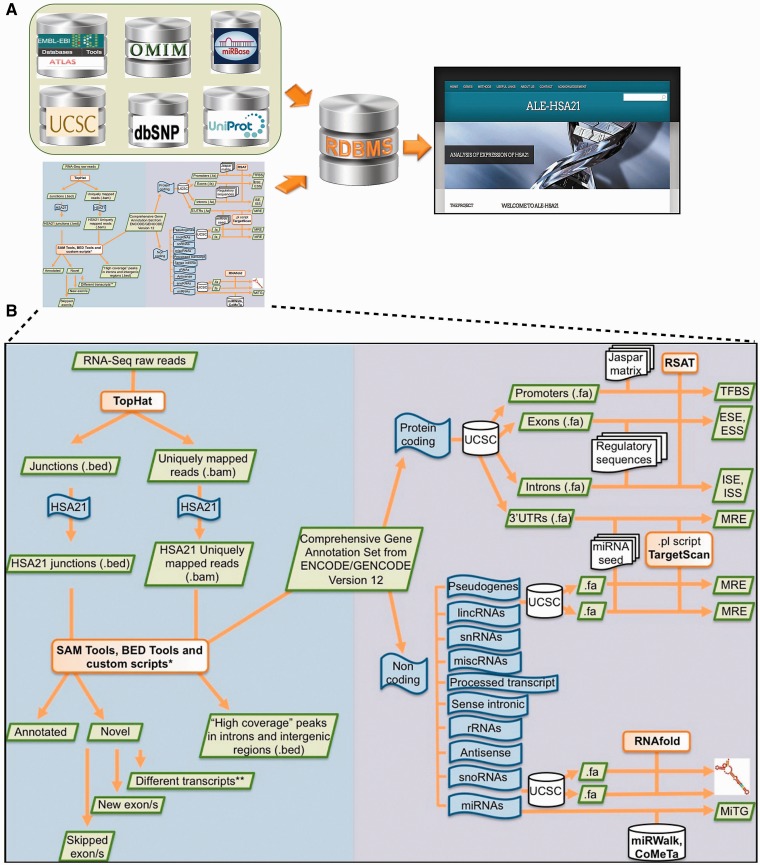
Schematic overview of data collected in ALE-HSA21 and of the computational analysis. Panel (**A**) shows a list of open-access databases used to retrieve information and the cartoon of the computational workflow used to analyze the data. Data derived from these sources were collected and integrated into our relational database and its web interface, represented on the right by the Homepage of ALE-HSA21. On the left part of panel (**B**) is schematically illustrated the computational approach used to analyze RNA-Seq data sets. In the right part it is depicted the workflow of the *in silico* analysis performed on the regulatory sequences for both coding and noncoding transcripts of chromosome 21. Green boxes indicate data files; in orange are indicated the computational tools used to perform the analysis; in blue are indicated the ‘features’ of interest; in white are indicated the databases and the regulatory data sets retrieved from them.

Nucleotide sequences for each promoter, exon, intron and 3′ UTR were downloaded in FASTA format from UCSC database. In detail, of 1702 HSA21 protein-coding transcripts, 1295 were exclusively annotated in the ‘Comprehensive Gene Annotation Set from GENCODE Version 12’, 139 only in RefSeq (release 56) and 268 in both of them. Simultaneously, 462 HSA21 noncoding transcripts were divided in 10 classes according to GENCODE v12 (Supplementary Table S1 and illustrated in the right panel of Figure 1B). For miRNAs, a comprehensive list of 30 entries was created merging information from different databases (GENCODE, RefSeq, Ensembl and miRBase). For the other noncoding transcripts, we retrieved from the ‘Comprehensive Gene Annotation Set from GENCODE v12’ a list of 220 long intergenic noncoding RNAs (lincRNAs), 24 pseudogenes, 15 sense-intronic transcripts, 80 antisense transcripts, 40 processed transcripts, 21 small nuclear RNAs, 19 small nucleolar RNAs (snoRNAs), 5 rRNAs and 8 miscellaneous RNA. Promoters' sequences (±1 kb from transcription start site) of coding transcripts were scanned for the presence of TF binding sites using the ‘matrix scan’ option of Regulatory Sequence Analysis Tools (RSAT) web server (17). Position weight matrices of 78 human transcription factors (TFs) were downloaded from JASPAR database. The ‘DNA pattern’ tool of RSAT web server was used to determine the presence of ‘consensus’ sequences for 106 exonic splicing enhancer and 50 silencer (ESE/ESS), 54 intronic splicing enhancer and 32 silencer (ISE/ISS). Regulatory sequences were downloaded from RegRNA web server (18). The number of analyzed exons and introns is shown in Table 1.

**Table 1.** Number of analyzed gene elements collected in ALE-HSA21 database

	Genes	Transcripts	Promoters	Exons	Introns	3′ UTR
Coding	238	1713	1713	14 177	12 454	1201^a^
Noncoding	296	462				244 ^b^
Total	534	2175	1713	14 177	12 454	1445

^a^The number of 3′ UTRs analyzed for the presence of MREs is smaller than the total number of transcripts because some of them lack UTR in the GENCODE annotation or are currently annotated as ‘processed transcripts’, ‘retained introns’ and other categories lacking a typical 3′ UTR.

^b^For noncoding RNAs, the entire sequences of 220 lincRNAs and 24 pseudogenes have been analyzed for the presence of MREs.

For the identification of miRNA responsive elements (MREs) within the mRNA 3′ UTRs, the complete list of 6121 mammalian miRNAs of TargetScan database was filtered for 4582 nonhuman miRNAs. Thus, targetscan_60.pl script downloaded from TargetScan was used to analyze 1201 3′UTRs of protein-coding transcripts, searching for binding sites of 1539 miRNAs. Of note, the number of 3′UTRs analyzed for the presence of MREs is smaller than the total number of transcripts because some of them lack UTR in the GENCODE annotation or are annotated as ‘processed transcripts’, ‘retained introns’ and other categories lacking the typical 3′UTR.

The same analysis was performed also to predict MREs within the entire nucleotide sequences of 220 lincRNAs' and 24 pseudogenes' transcripts mapping on HSA21.

RNAfold web server, with default parameters (19), was used to predict—and visualize—the secondary structure of 19 snoRNA and 30 pre-miRNA sequences (figures available on ALE-HSA21). Such analysis represents predictions of secondary structures based on minimum free energy and partition functions, and the images do not represent *in vivo* structures.

For the 19 HSA21 miRNAs annotated in miRBase (20), the mature miRNA sequence was also highlighted in such drawings. miRNA target genes (MiTGs) were predicted by miRWalk [using algorithms both for ‘validated’ and ‘predicted’ genes; (21)] and CoMeTa databases (22). Complete data were available for five miRNAs (miR99a, miR125b, miR155, miR802 and let-7 c). Finally, the three lists of MiTGs were intersected to determine a common pool using Venny (23).

### Database development and description

ALE-HSA21 is a database-driven Web site, more properly driven on a Relational Database Management System. It allows to structure the information contained in the web portal and to display them in tables. Overall, the database contains 534 HSA21 genes, consisting of 33.394 different genomic elements (Table 1). Database was implemented using server version 5.1.67—10.04.1 (Ubuntu)—and a web server Apache/2.2.14 (Ubuntu). MySQL client version 5.1.67—10.04.1 (Ubuntu)—and the free tool phpMyAdmin version 3.3.2 deb1ubuntu were used to handle the administration of MySQL over the World Wide Web.

### ALE-HSA21 Web site

The web-oriented side was created using the scripting language PHP. The Javascript technology for dynamic contents, the markup language HTML for static contents and style sheet CSS 2.0 were also used. All 3D images have been implemented using the ‘clickable image maps’ method, thus making only a certain portion of the image sensitive to mouse clicks, linking to different destinations and contents. The ALE-HSA21 code is validated according to the standard web of the international community W3C (World Wide Web Consortium: http://www.w3.org/) and therefore, although optimized for Mozilla Firefox, it is easily visible and accessible by all browsers and smartphones.

The web resource will be regularly updated on the basis of the progress of our study.

## Results

### Global organization of ALE-HSA21 web portal

ALE-HSA21 is an open-access user-friendly web resource built on a relational database. This web portal is a comprehensive ‘transcript-centric’ database for HSA21, in which data from different sources are dynamically integrated (Figure 1A). ALE-HSA21 contains 2164 annotated transcripts, generated through AS from 534 genes. Particularly, 1702 transcripts derive from 238 protein-coding genes and 462 from 296 noncoding genes (Table 1; Supplementary Figure S2). Moreover, ALE-HSA21 also collects 11 transcripts (of protein-coding genes) identified—and validated—through the computational reanalysis of our RNA-Seq data sets.

The web resource consists of five main sections, schematically shown in the block diagram of Supplementary Figure S3 and in the ‘Sitemap’ of ALE-HSA21. The core is represented by the sections ‘Coding genes’—divided in ‘Annotated’ and ‘Novel’—and ‘Noncoding genes’. The ‘Novel’ subsection hosts transcripts identified *de novo* by the analysis of our RNA-Seq data sets by merging custom computational workflows to open source tools and public databases (Figure 1B). In this subsection, only experimentally validated new HSA21 transcripts are reported. For all of them, sequences have been submitted and approved by EMBL Nucleotide Sequence Database.

Both sections, ‘Coding genes’ and ‘Noncoding genes’, contain two types of data: genomic/structural and regulatory. In particular, for all HSA21 transcripts, ALE-HSA21 provides a brief gene/category description, sequences in FASTA format and 3D structures. These dynamic images are linked to the regulatory data, consisting of computationally predicted motifs within nucleotide sequences. In addition, for coding genes, this web resource integrates links to public repositories of expression data, gene networks and ontologies, nucleotide variations, proteins and association to disease (Gene Expression Atlas, Gene Networks, dbSNP, UniProt and OMIM, respectively).

### *In silico* identification and experimental validation of novel HSA21 transcripts

Our RNA-Seq data sets (11) were reanalyzed using a custom computational workflow (see ‘Materials and Methods’ section; schematized in Figure 1B). Through this *in silico* approach we identified different intronic/intergenic regions possibly representing novel transcripts (Supplementary File S2). Additionally, we checked the presence/absence of intronic/intergenic HCPs in two independent RNA-Seq data sets from HeLa and K562 cell lines (24) and tumor samples (manuscript in preparation), observing these are not cell-specific. Similarly, for known HSA21 genes putative extended UTRs and novel splice junctions—according to our categorization method—were also detected (Supplementary Files S3 and S4). Filtering out signals (i.e. mapped reads) from pre-mRNAs and repeats, and considering their overlap with AceView predictions and/or expressed sequence tags, we confirmed the presence of 11 novel transcripts arising from six HSA21 genes (Table 2). Their *bona fide* was confirmed in progenitor cells used in our previous study (11), as well as in other cell lines (data not shown). Nucleotide sequences of these new transcripts were submitted to the EMBL Nucleotide Archive, and accession numbers are listed in Table 2. Newly identified transcripts were added to ALE-HSA21 database and included in the computational analyses, further described.

**Table 2.** Novel transcripts identified by RNA-Seq analysis

Gene symbol	Transcript	Accession number	Primer sequence (5′–3′)	
			Forward primer	Reverse primer
*IFNAR2*	IFNAR2_var1	HG380509	CTGGGAGTCCGCTTTCGTT	GGAGACTTTATTACTGCTTGC
*MCM3AP*	MCM3AP_HF584748	HF584748	AGTGCTGAGCGAACCGGAAG	GGCTCAACAGGAAATGGTAAA
*NRIP1*	NRIP1_HF584749	HF584749	GAGAGCTGCTGAAGAAGTAG	TAAATGAGAAAAAATGCATTGTC
*NRIP1*	NRIP1_HF584750	HF584750	GAGAGCTGCTGAAGAAGTAG	TAAATGAGAAAAAATGCATTGTC
*POFUT2*	POFUT2_var1	HG380510	GGGCCATGGCGACACTCA	TGTGTTTCTCAGCAGCAGGG
*POFUT2*	POFUT2_var2	HG380511	GGGCCATGGCGACACTCA	TTTATCCCTGGCGCTGCAC
*SAMSN1*	SAMSN1_all_skip	HG380514	GCACACTGCTGACTGTTTTC	ATCTTCCTCTCCTATTTGACG
*SAMSN1*	SAMSN1_var1	HG380512	GCACACTGCTGACTGTTTTC	ACTATAGAAGTGCTTGGTACT
*SAMSN1*	SAMSN1_var2	HG380513	GCACACTGCTGACTGTTTTC	ATCTTCCTCTCCTATTTGACG
*DYRK1A*	DYRK1A_var1	HF584751	TGTTATAGTTTTGCCGCTGGA	CTGTTGGTCACTTATGTTTGG
*DYRK1A*	DYRK1A_var2	HF584752	TGTTATAGTTTTGCCGCTGGA	CTGTTGGTCACTTATGTTTGG

Of note, potentially new transcripts reported in the Supplementary Files S2–S4, not yet validated by reverse transcriptase-polymerase chain reaction and Sanger sequencing, are, however, supported by RNA-Seq data (HCPs with uniquely mapped reads and/or a sufficient number of reads mapping on the splice junctions). However, as RNA-Seq data sets come from fragment libraries of 50 bp reads, and given the heuristic nature of algorithms for reads' alignment, despite our checks, false-positive alignments may have occurred.

### Computational analysis of regulatory sequences in protein-coding transcripts

**Figure 2.**
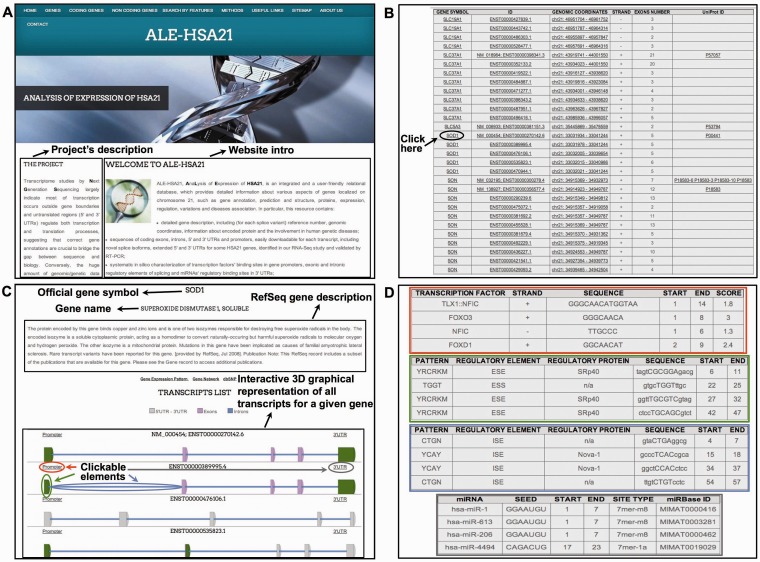
Screenshots from ALE-HSA21 web resource. Panel (**A**) shows the Homepage with Navigation Bar; panel (**B**) shows the list of HSA21 transcripts in the ‘Coding genes’ section in tabular format. Official gene symbol, ID, genomic coordinates, the sense of transcription, the number of exons and UniProt IDs are reported. The Black arrow and circle indicate an example of a clickable item (*SOD1* gene in the example). By clicking there, the users access the Gene Description page, depicted in Panel (**C**). Interactive 3D graphical representation for each transcript is embedded in this web page. Each gene element is linked to results of *in silico* analysis. Colored circles—red for ‘Promoter’, green for ‘exons’, light blue for ‘introns’ and gray for ‘3′ UTRs’—correspond to the clickable elements of the 3D images. The same color scheme is used in panel (**D**) to indicate the relative results for the computational analyses of those elements.

Predictive *in silico* analysis of regulatory sequences within gene promoters, exons, introns and 3′ UTRs was performed for both annotated and newly identified HSA21 protein-coding transcripts (Table 1). The ‘consensus’ sequences for 78 human TFs were predicted within gene promoters. Similarly to the RSAT output, the results are provided to the final user as tables with a ‘weight score’ column measured by the ‘Background model estimation method’ (17). Such tables, dynamically integrated on the web portal, can be accessed through the clickable ‘*Promoter*’ button in the 3D structure of each transcript (Figure 2C and D). In addition, to provide the users with ChIP-Seq (chromatin immunoprecipitation followed by massive sequencing) data for TFs of the ENCODE project, we also integrated a clickable button linked to these tracks, loaded into an open-access custom session of UCSC Genome Browser. This kind of approach, based both on computational predictions of TFs' binding motifs and experimental large-scale data, represents a starting point to investigate differential TFs' binding among distinct genes and, more interestingly, among different transcripts of the same gene.

Moreover, as ‘noncanonical’ exonic and intronic sequences are known to affect splicing—and mutations herein can cause monogenic or can be associated to complex disorders—we computationally predicted ESE/ESS and ISE/ISS within all exons/introns of each HSA21 transcript. Similar to the predictive analysis of TFs' binding sites, data in tabular format were included within ALE-HSA21 and dynamically integrated in 3D transcripts' models. The user can access these results by clicking the exon/intron of interest on the 3D structures (Figure 2C and D).

In addition, given the crucial role of miRNAs in the posttranscriptional regulation of mRNAs through their binding to 3′ UTRs (25, 26), we added to our resource this layer of gene regulation. Particularly, we computationally predicted the binding sites of all annotated human miRNAs within 3′ UTRs sequences of HSA21 mapping mRNAs. Results of such predictive analysis can be accessed through the ‘3′ UTR’ button integrated within the 3D graphical representation of each transcript (Figure 2C and D). Predicting the presence of differential MREs within distinct transcripts of the same gene can be rapidly and easily assessed browsing data of our computational analyses integrated in ALE-HSA21.

### Computational analysis for noncoding transcripts

**Figure 3.**
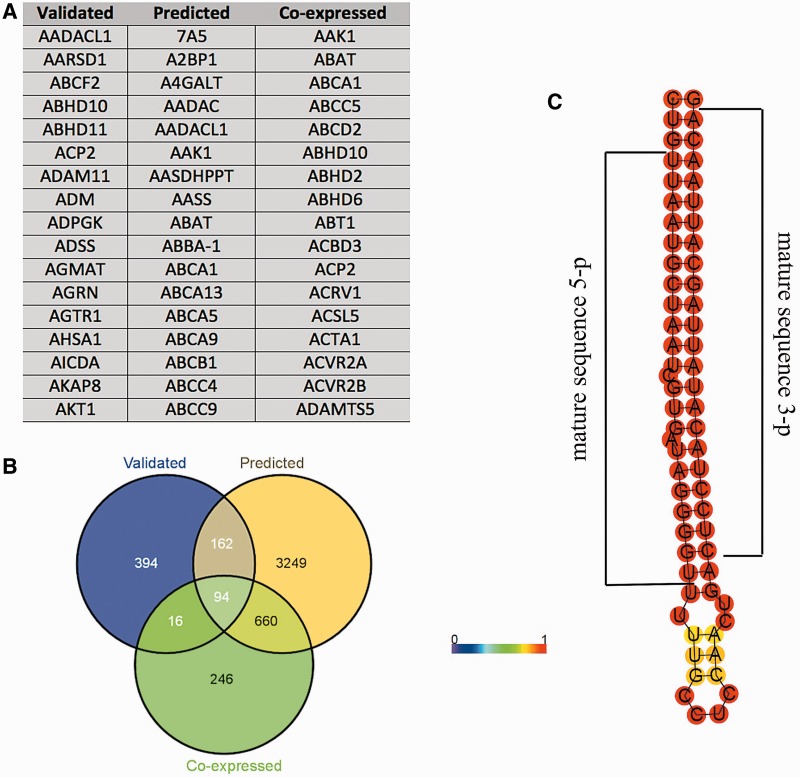
Example of the data provided for miRNAs in ALE-HSA21 web portal. In panel (**A**) and (**B**) are shown the results of the computational prediction of MiTGs in tabular format and Venn diagrams, respectively. Such data are accessible by clicking the ‘Target genes’ button embedded within miRNA web pages. ‘Validated’, ‘predicted’ and ‘co-expressed’ correspond to the target genes according to miRWalk and CoMeTa databases. Panel (**C**) shows a prediction of the secondary pre-miRNA structure obtained by RNAfold. Mature miRNA sequences are indicated by black brackets.

Recent evidences have shown that pseudogenes sequester miRNAs and act as competitive endogenous RNAs, and a similar mechanism has been proposed for lincRNAs (27). The role of these transcripts as miRNAs' sponges has been directly linked to carcinogenesis and muscle differentiation (28–30), and proposed for the onset of neurodegenerative diseases (31). Given these considerations, using prediction algorithms we searched for MREs in HSA21 lincRNAs and pseudogenes. The results were dynamically integrated in tabular format within the ‘Noncoding genes’ section of ALE-HSA21, accessible by clicking the ‘miRNA binding sites’ button located in the web page of the related transcript. In addition, using three different prediction algorithms, we also independently predicted—for the five HSA21 miRNAs available in CoMeTa and miRWalk databases—the putative MiTGs, not limiting such analysis to HSA21 genes. Data integrated in the web portal are provided in tabular format (Figure 3A). Moreover, the intersections between the three above-mentioned lists of computationally predicted MITGs—one for each prediction algorithm used—are shown as Venn diagrams (Figure 3B). These data allow rapidly observing a common pool of target genes, possibly regulated by the same HSA21 miRNA. Finally, as miRNAs and snoRNAs have a peculiar folding, which in turn determines their biological functions, we predicted their secondary structures and integrated these data as static images within ALE-HSA21 (Figure 3C). More in detail, in each miRNA and snoRNA drawing, nucleotides are colored by the base-pair probabilities, according to local measures of reliability, as described in (32). For all 19 HSA21 miRNAs, currently annotated in miRBase, we highlighted the nucleotide sequences corresponding to the mature—and functional—form of these miRNAs.

### Exploring ALE-HSA21

The intuitive interface of ALE-HSA21 makes this web resource an easy and fast—although scientifically accurate, comprehensive and updated—tool to retrieve, in few clicks, relevant information about HSA21 genes and transcripts. A detailed user's guide on how to browse ALE-HSA21 and how to extract useful information is provided as Supplementary File S5 and also available on the web portal. The homepage is shown in Figure 2A. From the navigation bar the user can access to the different sections of the portal. Within the ‘Coding genes’ section there is the complete and updated list of HSA21 transcripts, both known and newly identified in this study, in a user-friendly tabular format (Figure 2B). For each transcript, there is the official gene symbol, ID (NM and/or ENST according to the RefSeq and the GENCODE annotations), the genomic coordinates with a link to an open-access session of UCSC Genome Browser, ad hoc created with chromosome 21 coverage files, the sense of transcription (indicated by ‘+/-’ strand), the number of exons and the corresponding, if any, UniProt IDs. Each feature has a link to other sections of the web portal as well as to other external widely used databases. From the gene list table (Figure 2B) the user can easily access an interactive 3D graphical representation of all the transcripts. A ‘Search by Gene’ button allows a direct and quick access to all information for a given gene of interest. For each gene, such information consist of (i) full gene name and gene symbol, (ii) brief gene description with eventual literature references, (iii) links to gene expression data, gene networks and ontologies, (iv) single-nucleotide polymorphisms, (v) involvement in human diseases and (vi) 3D structures of all transcripts generated by AS (Figure 2C). In the 3D structures, each gene element (i.e. promoter, exons, introns and 3′ UTR) is clickable, allowing to directly access the results of our *in silico* analysis of regulatory sequences. As previously described, these data are provided in easily comprehensible tabular format, as illustrated in Figure 2D. In addition, for each element, below the results table, the nucleotide sequences are viewable and downloadable (FASTA format).

The ‘Noncoding genes’ section contains the comprehensive list of all HSA21 noncoding transcripts, divided in 10 categories, as depicted in Figure 1B. Similar to the ‘Coding genes’ section, by clicking on a specific transcript ID, the users can retrieve structural and regulatory data, described in the previous paragraph. In particular, for lincRNAs and pseudogenes, clickable ‘miRNA binding sites’ button allows accessing a complete list of predicted MREs in tabular format. Furthermore, the ‘Target genes’ button, included within the web pages dedicated to miRNAs, is directly linked to the results of our analysis on genes predicted to be targets of the selected miRNA.

Finally, a ‘Search by Features’ section has been implemented to ease the browsing ALE-HSA21 for specific features of interest, such as Gene Symbol, Transcript ID and UniProt ID.

## Conclusion

RNA-Seq studies have clearly shown that the vast majority of—if not all—human genes undergo AS, generating different transcripts (33, 34). These can be transcribed from alternative promoters and may possibly be regulated by different TFs as well as epigenetic factors (i.e. methylation and/or histone modifications). Transcripts with alternative 3′ UTRs may also undergo different co- and posttranscriptional regulation by miRNAs. In light of this, ALE-HSA21 is designed to provide, at the transcript level, computational predictions of TFs' binding sites in promoters as well as MREs in 3′ UTRs. Nonetheless, the presence of *in silico* predicted binding motifs does not guarantee a specific regulatory factor will bind that sequence. Future studies, based on ChIP-Seq data sets for TFs and sequencing of small RNAs, will surely allow us to improve the accuracy of this analysis. Understanding if alternative transcripts may be targets of different miRNAs, or if their transcription may be triggered by the same TFs, is clearly important to address their differential regulation, both in physiological and pathological conditions.

Moreover, it is known that different transcripts can arise from the same gene through the usage of alternative canonical splice sites, as well as noncanonical splicing enhancer/silencer sequences. Mutations within these regions affect splicing, causing diseases (35). Among them, familial isolated growth hormone deficiency type II is caused by different mutations occurring in the 5′ splice site, ISE and ESE that increase splicing of the exon 3 of *GH1* gene (36–38). In addition, aberrant splicing events—such as those caused by mutations in *WT1* gene in Frasier syndrome—may alter isoform abundance, affecting several cell processes (39–41). Thus, cataloguing the predicted binding sites for auxiliary splicing factors within all the splicing isoforms of a gene is an added value for clinicians studying human diseases.

Moreover, incomplete annotation of transcripts can lead to misinterpret the effect of nucleotide variations, both mutations and single-nucleotide polymorphisms. On the opposite, the exact knowledge of all splicing isoforms is crucial for clinicians to identify disease-causing mutations. The discovery of a 3′ terminal exon of *RPGR* gene—mutated in 60% of X-linked retinitis pigmentosa patients—is one of the first and most convincing examples of the link between AS and human diseases (6).

Such evidence highlights the importance to study at the isoform level, rather than at gene level, both physiological processes and disease mechanisms. The recently developed DataBase of Alternative Transcripts Expression (DBATE), valuable source of expression data for AS variants, is a good example (42).

In this ‘scenario’, our pilot transcript-centric database represents a fast and intuitive resource for medical geneticists interested in HSA21-related pathologies (such as DS), as well as for researchers investigating any molecular pathway or cell process involving HSA21 genes. Because ALE-HSA21 is an easy-to-use resource, it is accessible to all scientists with low experience in computational biology or informatics. ALE-HSA21 has been conceived to simply and rapidly provide the user with data usually dispersed in distinct databases or accessible by independently using different computational tools. The presence of genomic data, as well as of *in silico* predictions of regulatory sequences, links to gene expression, mutation and gene network databases in a unique Web site is an added value of ALE-HSA21.

Finally, our resource is in line with the growing interest for ncRNAs—supported by the wide diffusion of databases such as miRBase (20), miRWalk (21), lncRNome (43), lncRNA db (44), NONCODE (45) and Pseudofam (46). ALE-HSA21 provides open-access computational predictions about the presence of regulatory sequences for pseudogenes, lncRNAs and miRNAs, known to be involved in several biological processes.

This study is likely to represent an interesting proof-of-concept and a starting point for implementing similar resources with the aim to integrate information available in different databases to ‘omics’ data, generated by next-generation sequencing (RNA-Seq, ChIP-Seq and MeDIP-Seq) or by other large-scale technologies.
